# Sensitivity of Household Transmission to Household Contact Structure and Size

**DOI:** 10.1371/journal.pone.0022461

**Published:** 2011-08-01

**Authors:** Achla Marathe, Bryan Lewis, Jiangzhuo Chen, Stephen Eubank

**Affiliations:** 1 Network Dynamics and Simulation Sciences Lab, Virginia Bioinformatics Institute at Virginia Tech, Blacksburg, Virginia, United States of America; 2 Department of Agricultural and Applied Economics, Virginia Tech, Blacksburg, Virginia, United States of America; 3 Department of Physics, Virginia Tech, Blacksburg, Virginia, United States of America; University of Hong Kong, Hong Kong

## Abstract

**Objective:**

Study the influence of household contact structure on the spread of an influenza-like illness. Examine whether changes to in-home care giving arrangements can significantly affect the household transmission counts.

**Method:**

We simulate two different behaviors for the symptomatic person; either s/he remains at home in contact with everyone else in the household or s/he remains at home in contact with only the primary caregiver in the household. The two different cases are referred to as *full mixing* and *single caregiver*, respectively.

**Results:**

The results show that the *household’s cumulative transmission count* is lower in case of a *single caregiver* configuration than in the *full mixing* case. The household transmissions vary almost linearly with the household size in both *single caregiver* and *full mixing* cases. However the difference in household transmissions due to the difference in household structure grows with the household size especially in case of moderate flu.

**Conclusions:**

These results suggest that details about human behavior and household structure do matter in epidemiological models. The policy of home isolation of the sick has significant effect on the household transmission count depending upon the household size.

## Introduction

This paper aims to study the influence of household contact structure on the spread of an influenza-like illness. Public policy frequently recommends in-home care giving for those who are ill. This policy increases the risk of household transmission for the rest of the household members. Household transmissions can vary significantly depending upon contacts among the household members. We use simulations to assess the sensitivity of household transmission to contact structures within the household. Two extreme possibilities are considered for the structure of contacts among individuals within a household: *full mixing* and *single caregiver*. In the *full mixing* case every person (whether sick or not) is in contact with every other person in the household, as is often assumed in models with complete mixing [1], [2]. In the *single caregiver* case every symptomatic person is only in contact with a single primary caregiver (who, in turn, is in contact with every other person in the household), as is often recommended by public health agencies.

The *full mixing* structure is commonly used to model within-household contacts in network models for epidemiology [1]–[5]. The *single caregiver* contact pattern is suggested both by recent data on household secondary attack rates for H1N1[6] and by commonly practiced home care giving strategies[7]. For example, a child who becomes ill is often sequestered from the rest of the family except for a single adult who provides care.

## Methods

We simulate the spread of an influenza-like illness across two synthetic social networks representing the cities of Miami and Seattle, chosen for their dramatically different household size and age structures. The simulation is run using EpiFast, a fast agent-based epidemic simulation tool [8]. The disease model and the social network estimation are described in detail in the supporting information (appendix S1) and in peer-reviewed studies [3], [5], [9], [10]. An SEIR model is used to represent the disease progression within the host. For each individual, the incubation period duration is sampled from a discrete distribution with mean 1.9 days and standard deviation 0.49 day; the infectious period duration is sampled from a discrete distribution with mean 4.1 days and standard deviation 0.89 [1]. Half of infections result in identifiable symptoms, the other half are asymptomatic and are 47% less infectious than those with symptoms. Five infections from external sources occur within the population each day to seed the epidemic. The simulation is run for 300 days. Reported results are based on an average of 100 simulation replicates.

**Figure 1:**
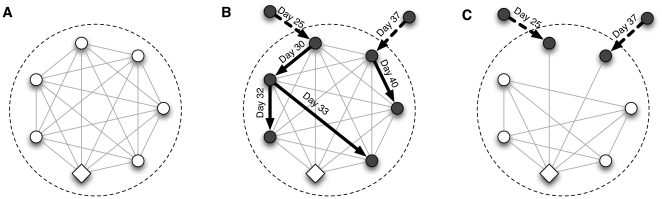
The contact structure within a household may change when a member becomes ill. The left panel shows a *full mixing* graph representing contacts between every pair of people in a household. Each node represents a member of the household and each edge represents a contact between them. The diamond shaped node represents the primary caregiver. Panel B in the middle shows that two members become infected from the outside contacts (nodes outside the big dashed circle). The index case spreads infection to 3 other members in the house which are marked by filled nodes; the second introduction infects one more member. Together they transmit to 4 members in the household so in this example, the transmission cumulative count is 4 and the proportion of infected members in the household under our definition is 4 out of 7 or 0.57. Panel C shows the contact pattern in a household with a *single caregiver*. The two infected nodes are now in contact with only the caregiver and no one else.

This paper defines household transmissions as all infections between household members. This includes all household members who are infected by another household member, even those that result from subsequent reintroductions of illness following the initial index case. Thus in the scenario illustrated in Figure 1b there are four household transmissions, caused by two introductions of illness, and in the scenario illustrated in Figure 1c there are zero household transmissions despite two introductions of illness. All infections are counted as household transmissions regardless of whether they are symptomatic or asymptomatic. This definition of household transmissions has been used in the literature before [11] although some researchers report cumulative secondary infections in the household and commonly impose a time limit of 7 to 14 days to restrict to observed infections that can be epidemiologically linked to the index case in the household [6], [12], [13]. We do not impose such a constraint because we can determine from the simulation whether an infection was caused by within-household transmission. Other measures of within-household transmission are of course possible, but we do not expect the results here to be sensitive to the choice of measure.

For all the simulations, symptomatic individuals undergo home isolation, modeled by removing all the non-home contacts for the individual. Thus they can only expose household members to infection. To study the effect of different household structures, we introduce two different behaviors for the symptomatic person: either s/he remains at home in contact with everyone else in the household or s/he remains at home in contact with only the primary caregiver in the household. The two different cases will be referred to as *full mixing* and *single caregiver*, respectively. We choose Miami and Seattle because these two cities have significantly different age and household size distributions as shown in Table 1. Seattle has a younger population than Miami but Miami has larger households than Seattle. When the effect of age based intervention is measured on the total attack rate in the two cities, these differences seem to cancel each other out in the *full mixing* case [14].

**Table 1:** Demographics of Miami and Seattle.

	Miami	Seattle
Total Population	2,095,627	3,211,727
Age Percentage in Each Category		
Preschool (0–4yrs.)	6.74	6.78
School Age (5–18 yrs.)	15.03	20.33
Adults (19–64 yrs.)	65.04	63.08
Seniors (65+ yrs.)	13.18	9.8
% Households (and % People) by Household Size		
1–2 persons	50.13 (26.99)	60.75 (37.65)
3–4 persons	34.04 (41.08)	29.91 (41.45)
5–6 persons	12.89 (23.92)	8.14 (17.09)
7 or more	2.94 (8.01)	1.2 (3.81)

Age and household size distributions of Miami and Seattle, based on 2000 US Census.

The overall attack rate is similar in these two cities because disease spreads more among children and among larger households due to the higher number of contacts. This research aims to examine the effect of home-isolation strategy on attack rate when the household structure is assumed to be a *single caregiver* rather than *full mixing*. We hypothesize that because the *single caregiver* structure reduces the number of household contacts, it will also reduce the attack rate.

In a *single caregiver* case, each symptomatic non-caregiver has only one contact (his caregiver), while in a *full mixing* case he has contact with all family members. We expect everyone’s likelihood of infection to decrease in a *single caregiver* case due to reduced contacts with infectious people. For a susceptible non-caregiver this is mainly because he has less contact with sick family members; for a susceptible caregiver this is because there are fewer sick family members. For simplicity of explication, the disease model does not include an age-dependent susceptibility. Possible consequences of such age-dependence are discussed in the Conclusions section.

Given the assumptions about behavior and those embedded in the disease model and social networks, the only free parameter is 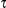, the rate at which disease is transmitted between an infectious person and a susceptible person when they are in contact. 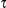 is expressed as a probability per unit of contact time. The overall infection attack rate in an epidemic is a function of 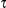 and the social network (and other parameters held fixed here). We compare and contrast the relationship between the attack rate and 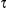 in 100 simulation runs for each of the two cities under both assumptions about household structure. We also compare the dependence of household transmission on household size with that observed for H1N1.

### Rules for Assigning Primary Caregiver in the Household

The study uses the following rules to designate the primary caregiver in each household:

•In a household with only one adult or with no adult, the oldest person is the primary caregiver.
•In a household with 2 or more adults.
  - If there is a non working female adult she is the primary caregiver, else the non working male adult is the primary caregiver. If there are multiple non-working females/males available, a random selection is made.
  - If all adults are working, oldest female is the primary caregiver.
  - If all adults are working and there are no females, the oldest male is the primary caregiver.

We do not intend to suggest this as an optimal or even good set of rules, but we believe it reflects common practice.

## Results

**Figure 2:**
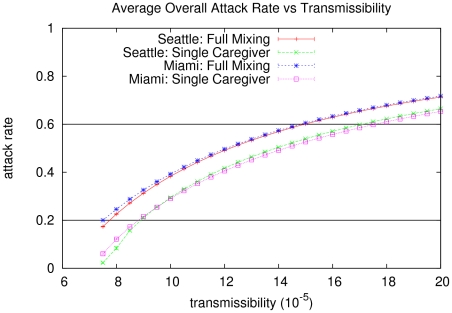
Mean overall infection rate (averaged over 100 runs) as a function of transmission rate 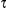 for Miami and Seattle in the *full mixing* and *single caregiver* case. The horizontal lines indicate the attack rates in Miami with the full mixing model under high and moderate levels of infectiousness, respectively.

Figure 2 shows the sensitivity of the overall attack rate to the transmissibility, 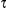, for the two cases studied in each city. The same 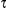 results in a higher attack rate in the *full mixing* case than in the *single caregiver* case for both Miami and Seattle.

Table 2 shows the overall proportion of the population infected under all 8 experimental conditions. We consider a factorial design with the following treatments: two levels of infectiousness, high and moderate (yielding attack rates of roughly 60% and 20%), in order to check the robustness of the results across different attack rates; two cities, to demonstrate the relative influence of demographic features of a population; and two household contact structures. When compared to the full mixing case, the single caregiver household contact structure results in a lower overall proportion of the population becoming infected. At the high levels of infectiousness there is a 13% and 10% reduction and for moderate levels of infectiousness there is a 70% and 88% reduction, for Miami and Seattle respectively. Note that even under identical household contact structures and levels of infectiousness, the overall attack rates differ between Miami and Seattle. There are several competing factors that influence these differences, but generally they result from demographic differences between the two cities and the effects of those differences on the social network of the cities themselves.

**Table 2:** Simulated infection attack rate by city, household structure, and transmissibility.

City	Household Structure	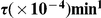	Proportion Infected
Miami	full mixing	1.50	0.61
Miami	single caregiver	1.50	0.53
Seattle	full mixing	1.50	0.60
Seattle	single caregiver	1.50	0.54
Miami	full mixing	0.75	0.20
Miami	single caregiver	0.75	0.06
Seattle	full mixing	0.75	0.17
Seattle	single caregiver	0.75	0.02

Relationship between the city, the household contact structure, the transmission rate (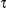) measured per minute, and the proportion of population infected. The higher value of 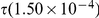 is the high level of infectiousness and the lower value 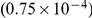 is the moderate level of infectiousness.

**Figure 3:**
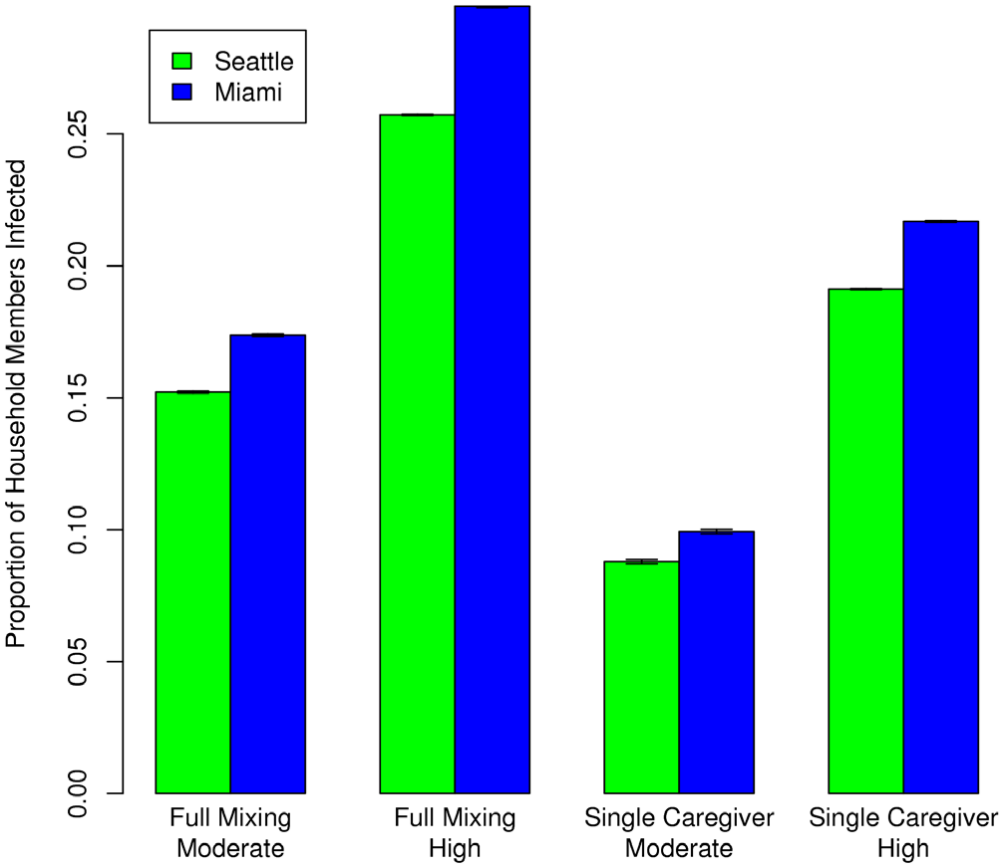
Proportion of household members infected (averaged over 100 runs and over all households) for Miami and Seattle. The x-axis shows the household structure and the overall attack rate (AR). This figure demonstrates that the *single caregiver* configuration indeed results in lower household transmission than the *full mixing* configuration.

Figure 3 shows a comparison of the proportion of infected members in a household for both cities, for *full mixing* and *single caregiver* cases, at two different levels of infectiousness. The proportion of infected members in the household is always higher in Miami than in Seattle. This is due to the fact that Miami has larger families which provide more opportunities for secondary and later infections when the sick person is kept at home. It also finds that the *single caregiver* case always results in fewer transmissions than the *full mixing* case in both cities.

**Figure 4:**
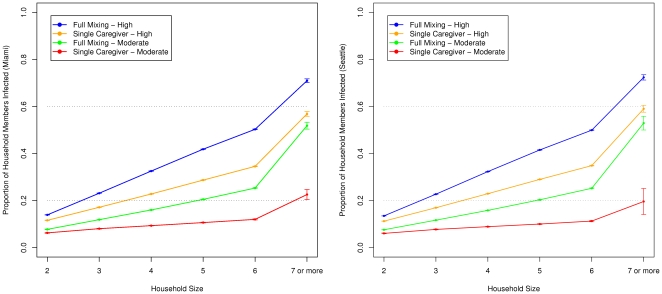
Proportion of the household infected vs. the household size. Left: for **Miami**; right: for **Seattle**. Households of size 7 and higher have been aggregated into one data point. As Table 1 shows, households of size 7 and higher constitute less than 3% of Miami households and 1.2% of Seattle households. The proportion of infected members in the household increases with the household size and is higher for the *full mixing* structure than for the *single caregiver* structure in both cities. The difference in proportion due to household structure also increases with household size; more so when the infectiousness is moderate.

Figure 4 compares the relationship between the proportion of household members infected (excluding the index case and the infections caused by outsiders) and the household size for the *full mixing* vs. *single caregiver* cases in each city for the levels of infectiousnsess. Note that transmission within the household can be lower or higher than the overall attack rate. Household transmissions capture only within-household infections (leaving out the index case and any infections caused by outsiders) whereas the overall attack rate measures all infections. As the household size increases, the proportion of household members infected also increases in both cities under both types of household structures. Results in Figure 4 from both Miami and Seattle show that household transmissions vary directly with the household size. The difference in transmissions due to household structure increases with the household size, especially when infectiousness is moderate.

**Figure 5:**
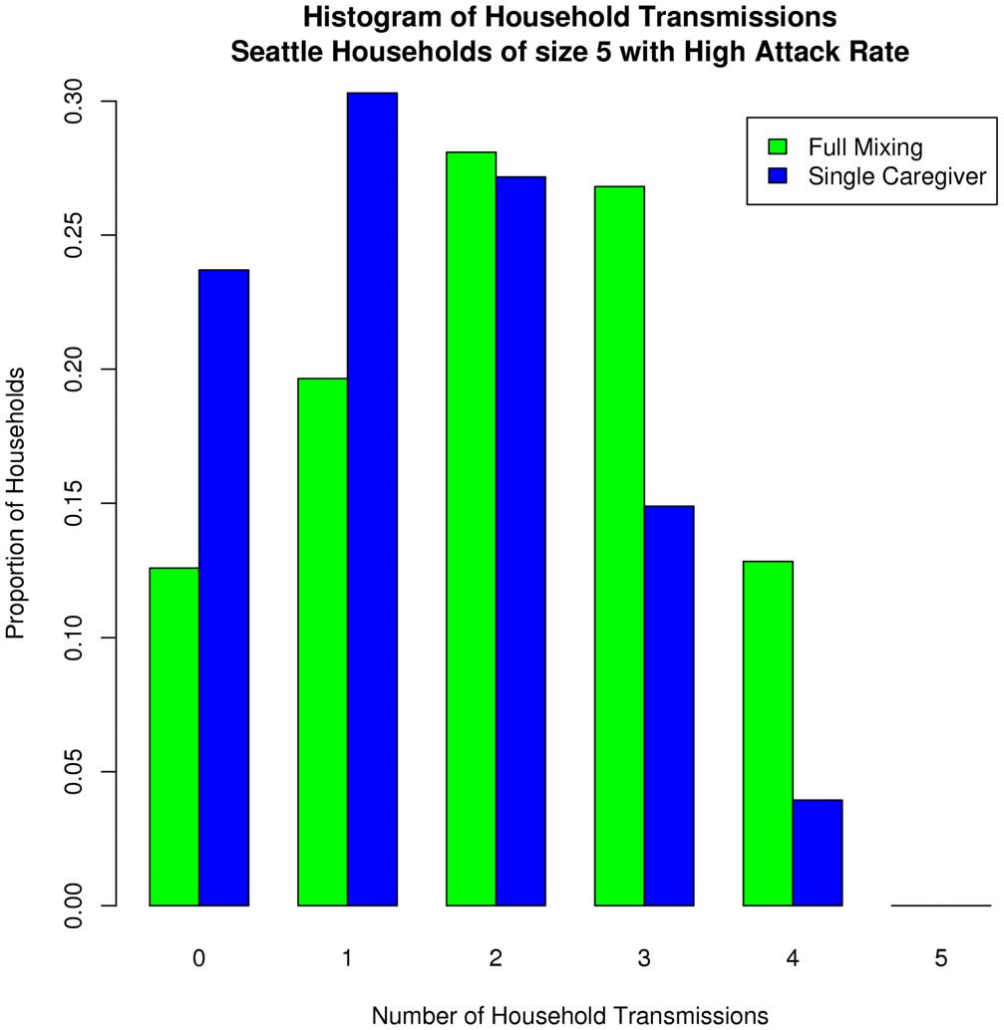
The distribution of infections within Seattle households of size 5. Under *full mixing* conditions more households experience higher transmissions within the household. In case of the *single caregiver* model a majority of households experience fewer than two transmissions within the household, predominantly the caregiver.

The stochastic variation across simulation runs is captured in the error bars (standard deviation) shown at the top of each panel. We also analyze the distribution of within-household transmissions for a given household size to capture the variation within a class of households. Figure 5 shows the results for households of size 5 in the city of Seattle with a high level of infectiousness. In the *full mixing* case, more households experience 2 or more transmissions within the household; in the *single caregiver* case, a majority of the households experience fewer than 2 household transmissions, predominantly to the caregiver.

## Discussion

The results argue for the importance of including detail about human behavior in epidemiological models, and for using appropriate disaggregated data for calibration. In particular, details about human behavior can lead to significant differences in inferring 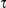 from an observed total attack rate. For the sake of argument, let us assume that the *single caregiver* case comes closer to representing reality than the *full mixing* case. This would imply that at the level of households, a model using *full mixing* within a household is incorrectly specified since it assumes higher mixing rates than the *single caregiver* case. Nonetheless, it is still possible to adjust or calibrate the *full mixing* model to any desired attack rate (often this process is erroneously considered to validate the model). However, the resulting inferred transmissibility will be systematically biased away from, and in this case downward from, its true value. When such a calibrated, but incorrectly specified model is used to study the effectiveness of interventions, two different problems arise:

1. The modeled effectiveness of interventions will be biased because the transmissibility is biased.

2. The modeled effectiveness of interventions that change the mis-specified part of the model will be wrong. For example, an intervention such as sequestration within a household will obviously have much less effect in the *single caregiver* case (presumed to be closer to reality) than in the *full mixing* case.

Relative rankings of intervention effectiveness may still be correct even though the absolute effectiveness is biased, but it depends on whether all interventions have similar sensitivity to the mis-specification.

It is important to note that the sensitivity to details of the model demonstrated here is not a problem peculiar to models that explicitly represent the details. In principle, there is a correspondence between highly detailed, or disaggregate, models and aggregate versions of those models with an *effective* interaction parameter that aggregates over the detailed interactions. For mathematical epidemiology, the most commonly used aggregate model is a compartmental model consisting of a set of nonlinear, coupled, first order ordinary differential equations. The coupling constants in these equations are effective interactions that aggregate the effects of the myriad pairwise transmissions simulated by a network model. Sensitivity to detail exists in the aggregate model – the effective interaction constant for an aggregate representation of the *full mixing* case is different from that for the *single caregiver* case. However, the sensitivity is hidden, because the process of aggregation requires making symmetry assumptions about the network.

Note that the relationship between household secondary attack rate and household size, as reported by [6] is negative. However there are significant differences between their study and this one. We use cumulative household transmissions whereas the authors in [6] consider only secondary infections. They use *real data* from spring of 2009 on 216 households and 600 contacts whereas this research uses *simulations* to analyze millions of contacts. In the spring of 2009, the H1N1 outbreak was quite mild compared to 60% and 20% overall infection rates assumed in this research. Also, in [6] the households may or may not have followed any interventions to contain the spread, whereas this study intervenes with two specific household behaviors. Under both the number of potential interactions between infectious and susceptible individuals within the household increase with the size of the household, explaining the increasing trend (especially since all within household transmissions are included). The goal of this research is to demonstrate that the structure of the household contacts has a significant influence on the epidemic. This kind of detailed analysis cannot easily be done with the data gathered from the real world.

We have explicitly ignored important effects such as age-dependent susceptibility. In actual use, such effects must be taken into account. We expect that they will interact in complicated ways with the household size effects modeled here, and provide yet another demonstration of the importance of tailoring intervention strategies to a region’s demographics.

### Conclusion

This research suggests that the policy of home isolation of the sick has significant implications for transmissions within the household. The impact of such a policy depends upon demographics such as household size and on details of human behavior (full mixing or partial mixing of the sick with the rest of the household members). Public health policy and recommendations should take this differential impact into account. Future research should consider the provision of prophylactic medicines to caregivers. Such a study could analyze the impact on both the overall attack rate and the caregiver’s relative risk.

## Supporting Information

Appendix S1 Detailed description of disease model and the social network construction.
(PDF)
