# Supplementary material for: Sensitivity of Household Transmission to Household Contact Structure and Size
Source: PLoS One. 2011 Aug 1;6(8):e22461. doi: 10.1371/journal.pone.0022461 (PMC3148222; doi:10.1371/journal.pone.0022461)
Supplement: Appendix S1 — Detailed description of disease model and the social network construction. (PDF) [file pone.0022461.s001.pdf]

Appendix S1

Construction of Synthetic Population and the Social Network

We have created synthetic social contact networks for several large regions in the US, representing the daily interactions between the inhabitants of the region. [1–4] The synthetic Seattle population has over 3.2 million individuals (represented as nodes in the contact graph), with over 88.7 million explicitly represented interactions (edges in the contact graph). The Miami population has nearly 2.1 million people and just over 52.7 million interactions. Our approach consists of four steps:

1. *population synthesis*, in which a synthetic representation of each household in a region is created from the US Census data;

2. *activity assignment*, in which each synthetic person in a household is assigned a set of activities to perform during the day, along with the times when the activities begin and end, as derived from the activity or time-use survey data from the National Household Travel Survey (NHTS), and American Time Use Survey (ATUS).

3. *location choice*, in which an appropriate real location is chosen for each activity for every synthetic person based on Dun & Bradstreet (D&B) data and land use data.

4. *social contact network*, in which each synthetic person is deemed to have made contact with a subset of other synthetic people simultaneously at the same location. The resulting social contact network is a graph whose vertices are synthetic people, labeled by their demographics, and whose edges represent contacts, labeled by duration of contact and type of activity.

The *population synthesis* step preserves the confidentiality of the individuals in the original data sets, yet produces realistic attributes and demographics for the synthetic individuals. Joint demographic distributions are reconstructed from the marginal distributions available in typical census data together with joint distributions in Public Use Microdata Samples (PUMS) using an iterative proportional fitting technique.

This technique guarantees that a census of our synthetic population is statistically indistinguishable from the input census when aggregated to block groups. Particularly important is step 2, i.e. *activity assignment*, in which each synthetic household is matched with one of the survey households, using a decision tree based on demographics such as the number of people in the household, number of children of various ages, household income, etc.

In the *location choice* step, for each household and each activity performed by this household, a preliminary assignment of a location is made based on observed land-use patterns, building capacity, tax data, etc. [5, 6]. Once activity based location assignments for each person in the population are made, we generate two types of graphs, a people-location graph G_{PL} and a people-people graph G_P.

A G_{PL} graph is constructed where P is the set of people and L is the set of locations. If a person $p \in P$ visits a location $\ell \in L$, there is an edge $(p, \ell, label) \in E(G_{PL})$ between them, where *label* represents the type of the activity and its start and end times. We also consider another graph G_P induced on the set of people: $(p_1, p_2) \in E(G_P)$ if there is a location $\ell \in L$ such that $(p_1, \ell), (p_2, \ell) \in E(G_{PL})$, and the time intervals during which p_1 and p_2 are present at ℓ overlap, i.e., there is a common location at which the two people p_1, p_2 are present at the same time.

Simulation of Disease Transmission

EpiFast is a very fast epidemic simulation tool. It achieves its speed by using a graph theory based algorithm (as opposed to event based simulation tools) and by efficiently dividing large social networks into many pieces so the computations can be done in parallel. This allows it to compute epidemics on

populations with 16 million individuals and includes their realistic person to person contact networks in less than 5 minutes (using a 96 node cluster). EpiFast also supports a range of interventions that can be dynamically defined (ie depend on the state of the epidemic rather than a fixed point in the simulation) which can rearrange the structure of social networks or change the degree of infectiousness or susceptibility of the individuals.

At its core, EpiFast represents each individuals disease state based on the commonly used SEIR (Susceptible, Exposed, Infectious, and Recovered) paradigm. Individuals move through these states and can infect their contacts while they are in the infectious state. EpiFast currently supports both a symptomatic and asymptomatic infectious state and allows for the asymptomatic state to have a different level of infectiousness. Additionally, the presence of symptoms can be used to identify individuals that are ill and thus can be have appropriate interventions applied to them based on this identification. A more precise and technical description of EpiFast's handling of disease follows.

Each person in the model is in one of the following four health states at any given time: *susceptible*, *exposed*, *infectious*, and *removed*.

- A person is in the susceptible state until he becomes exposed.
- If person v becomes exposed, he remains exposed for `Incub[v]` days (called *incubation period*), during which he is not infectious.
- Then he becomes infectious and remains infectious for `Infect[v]` days (called *infectious period*), during which he may be *symptomatic* or *asymptomatic*. An asymptomatic person is less likely to transmit the disease to other people.
- Finally he becomes removed (or recovered) and remains so permanently.

A contact network $G(V, E, w)$, is a directed, edge-weighted network. Each node corresponds to an individual in the population; each edge represents a contact between two end nodes during each day, and the edge weight is the contact duration. Edge (u, v) with weight $w(u, v)$ represents that node u has contact of duration $w(u, v)$ with node v every the day, during which the disease *may* transmit from node u to node v with probability $p(w(u, v))$.

On any given day, if a node u is in the infectious state, and v is in the susceptible state, then with probability

$$p(w(u, v)) = 1 - (1 - r)^{w(u,v)}$$

node u transmits the disease to node v on that day, where r is the probability of disease transmission for each unit of time of contact.

A crucial assumption made in almost all epidemic models is that of *independence*: we assume that the spread of infection from a node u to node v is completely independent of the infection from a node u' to node v. Similarly, an infected node u spreads the infection to each neighbor v, independent of the other neighbors of u.

References

1. Barrett C, Bisset K, Leidig J, Marathe A, Marathe M (2009) An integrated modeling environment to study the co-evolution of networks, individual behavior, and epidemics. AI Magazine 31: 75-87.

2. Bisset K, Marathe M (2009) A cyber-environment to support pandemic planning and response. DOE SciDAC Magazine : 36-47.

3. Eubank S, Guclu H, Kumar VSA, Marathe MV, Srinivasan A, et al. (2004) Modelling disease outbreaks in realistic urban social networks. Nature 429: 180-184.

4. Bisset K, Feng X, Marathe M, Yardi S (2009) Modeling interaction between individuals, social networks and public policy to support public health epidemiology .

5. Barrett C, Beckman R, Berkbigler K, Bisset K, Bush B, et al. (2001) TRANSIMS: Transportation analysis simulation system. Technical Report LA-UR-00-1725. An earlier version appears as a 7 part technical report series LA-UR-99-1658 and LA-UR-99-2574 to LA-UR-99-2580, Los Alamos National Laboratory Unclassified Report.

6. Beckman RJ, Baggerly KA, McKay MD (1996) Creating synthetic base-line populations. Transportation Research A – Policy and Practice 30: 415-429.
